# Effect of increasing doses of colchicine on the treatment of 333 COVID‐19 inpatients

**DOI:** 10.1002/iid3.1273

**Published:** 2024-05-26

**Authors:** Rumen Tiholov, Aleksander I. Lilov, Gergana Georgieva, Kiril R. Palaveev, Konstantin Tashkov, Vanyo Mitev

**Affiliations:** ^1^ Internal Medicine and Pulmology Department MHAT “Sv Ivan Rilsky” Kozloduy Bulgaria; ^2^ Department of Pneumology and Phthysiatrics SHATPPD “ Sofia district” Sofia Bulgaria; ^3^ Rheumatology Department MHAT Sv Ivan Rilsky Kozloduy Bulgaria; ^4^ Department of Social Pharmacy and Pharmacoeconomics, Faculty of Pharmacy Medical University—Sofia Sofia Bulgaria; ^5^ Department of Medical Chemistry and Biochemistry, Faculty of Medicine Medical University—Sofia Sofia Bulgaria

**Keywords:** colchicine, COVID‐19 mortality, COVID‐19 treatment, cytokine storm, NLRP3 inflammasome

## Abstract

**Background:**

Previous research done in Bulgaria demonstrated a fivefold reduction in mortality from COVID‐19 with increased doses of colchicine from two hospitals in the country. We report here a further 333 cases of COVID‐19 inpatients, treated with different doses of colchicine and its effect on mortality.

**Materials and Methods:**

A case‐control comparison from two additional hospitals was conducted between increased doses of colchicine and added bromhexine to standard of care (SOC) versus current SOC. Risk and odds ratio, as well as subgroup analysis, was conducted with newly reported data, alongside aggregate data from all hospital centers to determine the extent of mortality reduction in COVID‐19 inpatients.

**Results:**

There was a clear reduction in the mortality of inpatients with increasing doses of colchicine—between twofold and sevenfold. Colchicine loading doses of 4 mg are more effective than those with 2 mg. Despite these doses being higher than the so‐called “standard doses,” colchicine inpatients experienced lower mortality than SOC patients (5.7% vs. 19.53%). This mortality benefit was evident in different age subgroups, with a 4‐mg loading dose of colchicine proving slightly superior to a 2‐mg loading dose. Colchicine led to an overall relative risk reduction of 70.7%, with SOC patients having 3.91 higher odds of death. The safety of the doses was not different than the reported in the summary of product characteristics.

**Conclusion:**

Inpatients in Bulgaria with added colchicine and bromhexine to SOC achieved better clinical and mortality outcomes than those on SOC alone. These results question the World Health Organization—recommended strategy to inhibit viral replication. We posit that our treatment strategy to inhibit the Severe acute respiratory syndrome coronavirus 2 entry into the cell with inhaled bromhexine and the hyperactivated NLRP3 inflammasome with higher doses of colchicine, prevents the development of cytokine storm. The timing of the initiation of treatment seems critical.

## Graphical abstract

We present 333 cases treated with increased doses of colchicine, provided by different hospital centers. The result clearly shows that at higher, nonlife‐threatening doses of colchicine, the mortality rate drops sharply.

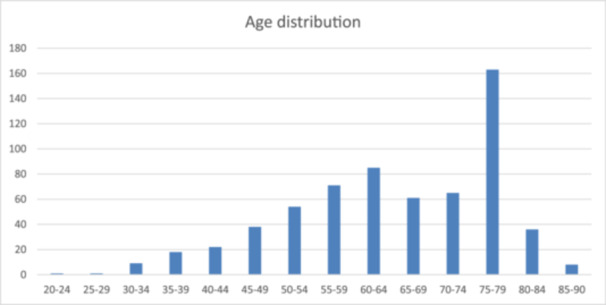

## 1 INTRODUCTION

Colchicine interferes with several inflammatory pathways involved in the pathogenesis of COVID‐19, including adhesion and activation of neutrophils, NLRP3 inflammasome activation, and cytokine release.^1^ It is now well known that overactivation of NLRP3 inflammasomes can promote inflammation process and aggravate COVID‐19, causing a cytokine storm (CS) and multiple organ dysfunction.^2^, ^3^

This is the reason why over 50 observational studies, randomized clinical trials, small randomized noncontrolled trials, and other research tried colchicine to treat COVID‐19.^4^ However, colchicine healing effect on COVID‐19 is subject to conflicting, and rather disappointing results.^5^ In all of these studies, loading doses of colchicine did not exceed 2 mg. It is surprising how persistently researchers attempt other clinical trials with the same low doses of colchicine, expecting a different result.^6^ This is likely due to the result obtained by the AGREE trial in 2010, which investigated colchicine for treatment of gout flare up where low doses of the drug (1.8 mg) were shown to be as effective as high doses (4.8 mg). This level of efficacy was sufficient for that particular trial, where the benefit to risk of adverse event ratio was important, and in which as many as 76.9% of patients developed diarrhea.^7^

It should be noted that the pathogenesis of COVID‐19 is different from that of gout. The COVID‐19 CS is generated in cells of the myeloid lineage,^8^ with the NLRP3 inflammasome being largely expressed in those cells.^9^ NLRP3 inflammasome can be inhibited by clinically supratherapeutic levels of colchicine.^10^, ^11^ However, leukocytes accumulate colchicine and its concentration in neutrophils may be more than 16 times the peak concentration in plasma.^12^

This suggests that clinically available colchicine doses may still be sufficient for the purposes of NLRP3 inflammasome inhibition.^12^, ^13^ The other possible reason for using low doses of colchicine is the fear of colchicine intoxication.^14^, ^15^ A careful analysis of the literature from 1947 until now shows that doses below 0.1 mg/kg are absolutely safe and, excluding cases of drug interactions and damaged liver and kidneys, no fatality has been described below 0.2 mg/kg.^16^

Thus, from the beginning of 2020, we started to administer higher doses of colchicine, which we found to prevent the COVID‐19 CS.^17^ Taking into account the above‐mentioned information, our therapeutic regimen consistent of loading doses between 0.04 and 0.045 mg/kg, but not more than 5 mg/day and this led to a fivefold decrease in the mortality of 452 inpatients.^6^

We now present another 333 cases treated with increased doses of colchicine, provided by other hospital centers. The result clearly shows that at higher, nonlife‐threatening doses of colchicine, the mortality rate drops sharply.

## 2 MATERIALS AND METHODS

We conducted a case‐control comparison between two groups of high‐risk COVID patients, presenting to a hospital, treated either with standard of care (SOC) at the admitting hospital, or a combination therapy of colchicine, and inhaled bromhexine hydrochloride (BHH) added to SOC. In Bulgaria, patients presenting to hospital are considered high‐risk patients according to the following characteristics: age over 65, at least one comorbidity—obesity; diabetes; chronic obstructive pulmonary disease (COPD); any cardiovascular disease; hypertension, and so forth.

Previously published results from hospital centers I and II^6^ are combined in this analysis with additional data from hospital centers III and IV. Previous data and analysis explored the effectiveness of higher loading doses in patients, presenting with severe COVID symptoms and at high risk versus SOC. Subsequently, two other hospital centers presented data from 2020 to 2021 with their own therapeutic regimens and mortality data, starting with a 2 or 4 mg colchicine loading dose + SOC versus SOC alone.

The main aim of the analysis was to first compare and analyze the data from hospital III (collected between November 6, 2020 and November 31, 2021) and hospital IV (collected between February 14, 2021 and March 7, 2022) independently, and afterward combine them with previously published data from hospitals I and II.

### 2.1 Patient collection and informed consent

The use of up to 5 mg colchicine for treating COVID‐19 patients was approved by the Medical Control Commission, University Hospital “Aleksandrovska,” Medical University—Sofia (Decision #17‐3‐54‐2020). Patients, admitting to the hospitals, signed a standard agreement to treatment form. Additionally, patients were informed of the potential side‐effects of colchicine treatment and the associated risks.

### 2.2 Statistical analyses

The calculated necessary sample size with a 95% confidence level and 5% margin of error was 289 patients, thus we set out to collect a minimum of 300 case group patients, ultimately collecting 333 from both hospitals, and 299 SOC patients.

All data were analyzed through MedCalc Statistical Software Version 22.0014 (© 2023 MedCalc Software Ltd.), and were additionally verified through Microsoft Excel calculations. Hospitals provided aggregate data on number of patients treated, therapeutic scheme, and mortality numbers. Relative risk (RR) and odds ratios (ORs) were calculated, based on the provided information. Fisher's exact test was additionally used to estimate the significance level of a 2 mg loading dose versus a 4 mg loading dose on mortality.

Two main research questions were postulated and the result calculated:

- The RR reduction in mortality and OR between combination therapy colchicine, inhaled bromhexine + SOC versus SOC alone in hospital centers III and IV.
- RR reduction in mortality and OR between higher and lower loading doses of colchicine, included in the therapeutic regimen. The main question investigated in this comparison was if a 4 mg loading dose on Day 1 resulted in better mortality outcomes than a 2 mg loading dose.

### 2.3 Therapeutic regimens

SOC usually comprises: Antibiotic treatment, corticosteroid, symptomatic relief medication, oxygen therapy, saline solutions. These medications were given as needed, per the discretion of the attending physician.

Hospital I and hospital II regimens have already been published and are not an object of analysis.^6^

#### 2.3.1 Hospital III

- SOC: Fraxiparine, antibiotics, corticosteroids, IV glucose/saline solutions, oxygen therapy, symptomatic relief medication.
- Colchicine 4 × 1 (2 mg)—4 days; 3 × 1(1.5 mg)—4 days; 2 × 1 (1 mg) up to Day 15 + inhalatory BHH.
- Colchicine 4 × 2 (4 mg)—4 days; 3 × 2(3 mg)—4 days; 2 × 2(2 mg)—4 days; 2 × 1(1 mg) up to Day 15 + inhalatory BHH.

#### 2.3.2 Hospital IV

- SOC: Fraxiparine, antibiotics, corticosteroids, IV glucose/saline solutions, oxygen therapy, symptomatic relief medication.
- Colchicine Day 1—4 mg; Day 2—3.5 mg; Day 3—3 mg; Day 4—2.5 mg; Day 5—2 mg; Day 6—1.5 mg; Day 7—1 mg up to Day 30 + inhalatory BHH.

Dose and frequency of BHH were the same between the colchicine 4 and 2 mg groups.

## 3 RESULTS

### 3.1 Baseline characteristics of patients

Table 1 shows the baseline characteristics in both centers of patients upon admission. Hospital IV had a lower mean age (61 years) compared to hospital III (68), leukocytes and creatinine levels, which are prognostic markers for mortality and complication, are both lower in hospital IV compared to III which might explain some of the mortality numbers seen later. Patients had at least one comorbidity with the most often seen comorbidities were hypertension (88%), diabetes (46%), and obesity (32%).

**Table 1:** Baseline characteristics of patients at admission.

Characteristic	Hospital IV				Hospital III			
Parameter and reference value	Mean (95% CI)	SD	Lowest value	Highest value	Mean (95% CI)	SD	Lowest value	Highest value
Age	61 (59–62)	13.31	22	86	68.8 (65.3–72.2)	14.35	37	94
Sex (%)	55% male 45% female	–	–	–	54% male 46% female	–	–	–
Leukocytes (3.5–10.5 × 10^9^/mm^3^)	10.73 (9.65–11.43)	3.57	4.2	25.4	17.04 (8.5– 25.54)	2.6	3.3	26.8
Creatinine (44–80 µmol/L)	88 (82–95)	39.153	50	405	96 (91–116)	52.23	65	162

Abbreviation: CI, confidence interval.

### 3.2 Hospital III data analysis

According to data, provided by hospital III, the average mortality for the SOC group, for both years, was 26.57%, whereas the average mortality in both colchicine groups was, on average, 10% (*p* < .0001). When comparing mortality percentages in the 2 versus 4 mg loading dose, a lower percentage was observed in the 4 mg group (7.5% vs. 11.25%, respectively), with a corresponding *p* value of .49.

Analyzing the RR and OR from Table 2 data, the colchicine group combined, regardless of loading dose, had a 62.4% reduction in mortality versus SOC (RR = 0.376; 95% confidence interval [CI] = 0.235–0.604), with a significance level of *p* < .001. Patients on SOC had 3.25 higher odds of experiencing a lethal outcome (OR = 3.257; 95% CI = 1.857–5.712), which was also statistically significant *p* < .0001.

**Table 2:** Hospital III data by year.

Year	2020			2021			
Therapeutic regimen	With colchicine 4 × 1 tabl. and nebulizations with bromhexine	SOC	Total	With colchicine 4 × 1 tabl. and nebulizations with bromhexine	With colchicine 4 × 2 tabl. and nebulizations with bromhexine	SOC	Total
Alive	53	48	101	89	74	57	220
Deceased	7	14	21	11	6	24	41
Total	60	62	122	100	80	81	261
Mortality (%)	11.7%	22.6%	17.3%	11%	7.5%	29.6%	15.7%

Colchicine 2 mg loading dose also showed a statistically significant reduction in mortality—57.7% (RR = 0.423; 95% CI = 0.253–0.707; *p* = .001), with patients on SOC having 2.85 higher odds of a lethal outcome (OR = 2.855; 95% CI = 1.54–5.28; *p* = .0008).

Colchicine 4 mg loading doses showed a more pronounced impact with a 71.8% reduction in the moratility (RR = 0.282; 95% CI = 0.125–0.638; *p* = .0024).

Overall data from hospital III shows an improved survival benefit of colchicine patients versus SOC, with a 4 mg loading dose showing improved survivability of patients.

### 3.3 Hospital IV analysis

Hospital IV considered only higher loading doses of colchicine (Table 3) for the active group. Observed mortality was 14.7% in the SOC group vs 2.1% in the colchicine group. RR calculations showed an 85.4% reduction in mortality (RR = 0.146; 95% CI = 0.035–0.604; *p* = .008), with patients in the SOC group experiencing a 7.86 times higher odds of death (OR = 7.868; 95% CI = 1.814–34.198; *p* = .0059).

**Table 3:** Hospital IV aggregate data.

Therapeutic regimen	SOC + C (4 mg)	SOC
Alive	91	133
Deceased	2	23
Total	93	156
Mortality (%)	2.2%	14.7%

Abbreviation: SOC, standard of care.

### 3.4 Combined analysis between hospitals III and IV versus SOC

**Figure 1:**
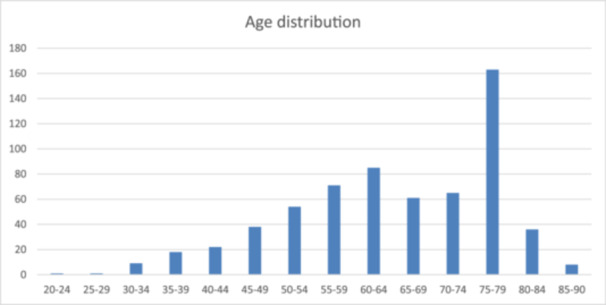
Histogram with an age distribution of admitted patients.

The average age of patients was 62.48 years; however, there was wide variability, with the youngest admitted patient being 22 years (hospital IV), and the oldest 94. The overall median age was 68 years, with the coefficient of skewness leaning toward older admitted patients (Figure 1). Gender distribution was equal with 55.9% men and 44.1% women. Table 4 shows the overall mortality stratification by age group in both the active and control groups. The average duration of hospital stay was 9.6 days (lowest 3, highest 37). For both groups, mortality was highest in the age bracket 75–79 (4.2% vs. 6.8%, respectively). Regrettably, the number of patients in each bracket was low to do comparisons of the proportions. However, some notable aspects are age groups 60–64, where no mortality was observed for the active group versus 1.2% in the control group; as well as age groups under 50, where there was no mortality in the active group, whereas there was some mortality observed in the control group. Total mortality was 20.4% versus 7.7% for the control and active groups, respectively.

**Table 4:** Mortality stratified by age.

Age	SOC + C (*N*)	Mortality, *N* (%)	SOC (*N*)	Mortality, *N* (%)
20–24	–	– (0%)	1	– (0%)
25–29	–	– (0%)	1	– (0%)
30–34	6	– (0%)	3	– (0%)
35–39	9	– (0%)	9	– (0%)
40–44	9	– (0%)	13	1 (0.33)
45–49	17	– (0%)	21	2 (0.66%)
50–54	25	2 (0.6%)	29	5 (1.68%)
55–59	32	2 (0.6%)	39	5 (1.68%)
60–64	48	– (0%)	37	9 (3.01%)
65–69	35	2 (0.6%)	26	5 (1.67%)
70–74	30	4 (1.2%)	35	8 (2.68%)
75–79	99	14 (4.2%)	64	20 (6.69%)
80–84	19	1 (0.3%)	17	4 (1.34%)
85–90	4	1 (0.3%)	4	2 (0.66%)
Total	333	26 (7.7%)	299	61 (20.4%)

The high mortality in the age groups 70+ is a potential bias indicator, since the average age for the country is 74 years. Several separate analyses were attempted to control for this bias:

- Average hospital stay for the colchicine and SOC group.
- Analysis of average O_2_ both the colchicine and control group.
- Analysis of average O_2_ saturation, in both deceased and surviving patients for SOC and SOC + C.

Among surviving patients, the average duration of hospital stay for the colchicine group was 10.44 days (SD = 4.7895), whereas for the control group average stay was 9.15 days (SD = 3.7702). *t* test for proportional differences suggests this difference was statistically significant (*p* = .0192)

Average O_2_ saturation for the colchicine group was 96.19 (SD = 1.41) versus 95.652 (SD = 2.1084) for the control group; difference = 0.5322 (*p* = .0316).

Average O_2_ saturation in the colchicine group, which survived was 96.86, whereas the average saturation for deceased patients in the active group was 93.53. Conversely, O_2_ saturation for control patients was 95.50 for surviving and 89.37 for deceased patients. The differences were significant, although the small increment between comparisons.

We posit that colchicine prolonged the duration of hospital stay, for seriously ill patients, while at the same time maintaining good saturation levels and preventing mortality. Conversely hospital stay was shorter for the control group due to increased mortality, and saturation levels dropped, contributing to the risk of death.

Each hospital independently has provided a statistically significant data set to observe a reduction in mortality in the case group. The pooled effect among 333 colchicine‐treated patients versus 299 SOC‐treated patients (hospital III + IV) showed a 63% reduction (RR = 0.37; 95%CI = 0.24–0.57; *p* < .001) and an RR reduction of 1 − RR = 0.628. The odds of experiencing a lethal outcome in the SOC group were 2.61 times higher (OR = 2.612; 95% CI = 1.609–4.243; *p* = .0001).

Overall, this confirms the added benefit of colchicine to SOC, we aimed additionally at calculating the effect estimate of the loading dose, based on the data provided by the two hospitals. In both centers, a total of 160 patients received a loading dose of 2 mg versus 173 with a loading dose of 4 mg (Table 5).

**Table 5:** Pooled mortality data of colchicine loading doses 2 versus 4 mg.

Initial loading dose	2 mg (hospital III)	4 mg (hospitals III + IV)
Alive	142	165
Deceased	18	8
Total	160	173
Mortality (%)	11.25%	4.62%

*Note*: Relative risk (RR) = 0.411 (95% confidence interval [CI] = 0.183–0.919, *p* = .03). A 59% mortality benefit was observed with a relative risk reduction of 1 − RR = 0.589. In general, patients with a higher loading dose have a higher probability of survival than patients on the 2 mg loading dose, who experience 2.61 higher odds of death (odd ratio = 2.614; 95% CI = 1.103–6.194, *p* = .029).

### 3.5 Pooled analysis from all hospital centers

Although these data are indicative of improved probability of survival, we aimed at narrowing the effect estimate by combining these data, with the previously published data from hospitals I and II. Among all patients, a total of 785 received colchicine (either with 2 and 4 mg loading dose) versus 512 on SOC (Table 4). A total of 625 of them received a 4 mg loading dose, 160 a 2 mg loading dose, and 473 were on SOC (Table 6).

**Table 6:** Pooled data for all colchicine‐treated patients.

Treatment	Colchicine (2 or 4 mg loading dose), BHH	SOC	Risk calculation	
Alive	740	412	RR = 0.293	OR = 3.991
Deceased	45	100	95% CI = 0.21– 0.41	95% CI = 2.75–5.79
Total	785	512	*p* < .0001	*p* < .0001
Mortality (%)	5.7%	19.53%	RRR = 1 − 0.293 = 0.707	

*Note*: Risk ratio and ORs continue to demonstrate a statistically significant survival benefit for the patients on colchicine than those on SOC, with relatively concise confidence intervals.

Abbreviations: BHH, bromhexine hydrochloride; CI, confidence interval; OR, odds ratio; RR, relative risk; RRR, relative risk reduction; SOC, standard of care.

Comparing the pooled 4 mg colchicine group vs SOC shows a RR of 0.20 (95% CI = 0.1359–0.3073, *p* < .0001) and a RR reduction of 0.80. OR = 5.937 (95% CI = 0.1359–0.3073, *p* < .0001). These results clearly show that patients on SOC have 5.9 times higher odds of death, while the 4 mg dose cohort shows an 80% survival probability.

Comparing the 4 versus 2 mg loading dose shows a RR of 0.384 (95% CI = 0.217–0.6794, *p* = .001), and a RR reduction of 1 − RR = 0.616. The odds of experiencing a lethal outcome are 2.80 times higher (OR = 2.807, 95% CI = 0.217–0.6794, *p* = .0012) (Table 7).

**Table 7:** Pooled data, separated by initial loading dose.

Treatment	Colchicine (4 mg loading dose), BHH	Colchicine (2 mg loading dose), BHH	SOC
Alive	598	142	373
Deceased	27	18	100
Total	625	160	473
Mortality (%)	5.7	11.25	21.14

Abbreviations: BHH, bromhexine hydrochloride; SOC, standard of care.

Mortality and survival numbers are given separately for each hospital center in Table 8. In total, between all four participating institutions, 625 patients received an initial colchicine loading dose of 4 mg, 160 patients received an initial loading dose of 2 mg, while the number of patients in the control group, receiving SOC, was 512 patients.

**Table 8:** Aggregate overview of the number of cases and control in each hospital for years 2020 and 2021.

	Hospital I		Hospital II		Hospital III			Hospital IV	
Loading dose	4 × 2 (4 mg)	SOC	4 × 2 (4 mg)	SOC	4 × 1 (2 mg) Day 1	4 × 2 (4 mg) Day 1	SOC	4 × 2 (4 mg)	SOC
Alive (*N*)	75	116	358	58	142	74	105	91	133
Deceased (*N*)	3	26	16	13	18	6	38	2	23
Total	78	142	374	71	160	80	143	93	156
Mortality (%)	3.2	18.3	4.2	18.3	11.25	7.5	26.57	2.1	14.74

Abbreviations: BHH, bromhexine hydrochloride; SOC, standard of care.

## 4 DISCUSSION

### 4.1 Two different strategies for treating COVID‐19

**Figure 2:**
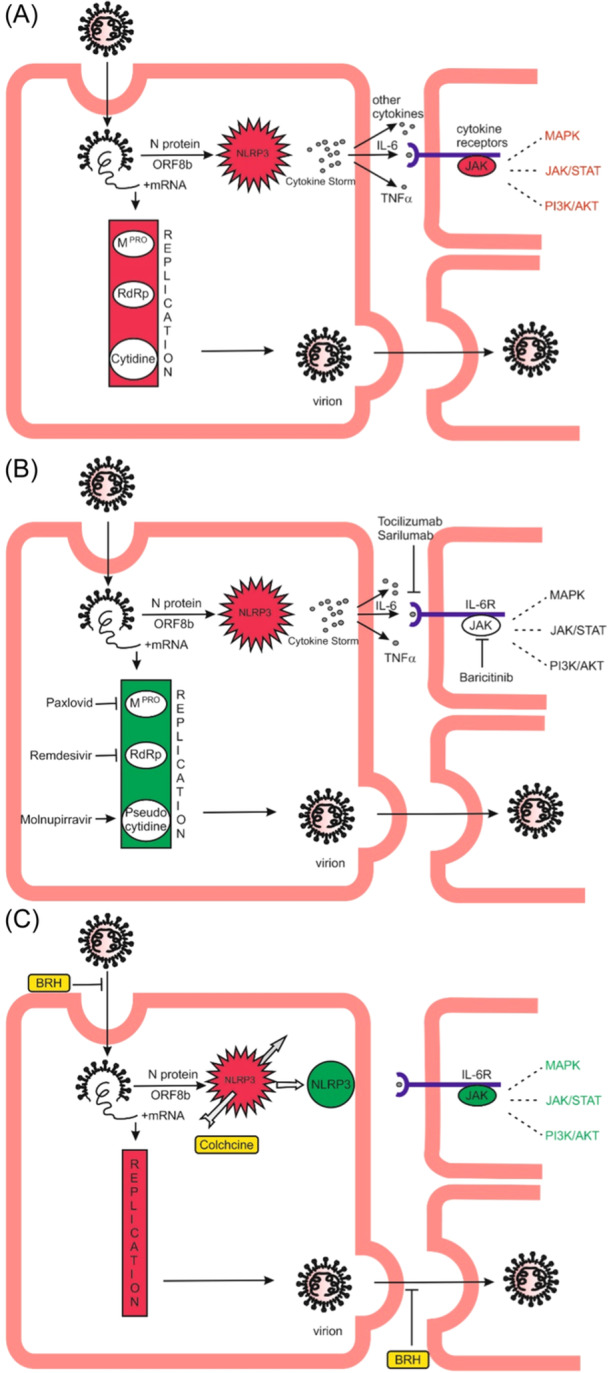
Two strategies for treating COVID‐19. (A) SARS‐CoV‐2 enters the cell mainly with the help of TMPRSS2. After virus replication, the virions infect new cells by the same mechanism. SARS‐CoV‐2 can directly or indirectly activate the NLRP3 inflammasome which is responsible for the CS, multiorgan damage, and death. (B) WHO recommends drugs that inhibit SARS‐CoV‐2 replication, the IL‐6 receptor, and the JAK/STAT pathway. However, there is no direct link between viral replication and NLRP3 inflammasome hyperactivation. At the same time, the NLRP3 inflammasome remains abnormally activated, and the level of dozens of cytokines high. This also explains why this expensive treatment with many side effects is partially successful. It is logical to assume that if the administration of antireceptor antibodies or JAK inhibitor is stopped before the normalization of the NLRP3 inflammasome/CS this could have fatal consequences for the patient. (C) Our strategy is to inhibit viral entry and spread with inhaled BHH and block the NLRP3 inflammasome hyperactivation with higher but safe doses of colchicine. BHH is most effective when taken prophylactically; when someone has been in contact with an infected or sick person, BHH inhalations should be started immediately and the effect is very good; the COVID‐19 patient should immediately start BHH inhalations with good effect, because the spread of the virus from cell to cell will be limited; in hospital conditions, inhalations with BHH will be useful, although there, in the first place, the question of inhibition of NLRP3 inflammasome is leading.^18^ After inhibition of NLRP3 inflammasome hyperactivation by colchicine, the CS is prevented, and blocking cytokine receptors and/or the JAK/STAT pathway becomes irrelevant. Time is crucial for the outcome of this very cheap treatment. AKT, protein kinase B; BHH, bromhexine hydrochloride; CS, cytokine storm; IL‐6, interleukin‐6; JAK, Janus kinase; MAPK, mitogen‐activated protein kinase; mRNA, messenger RNA; PI3K, phosphatidylinositol‐3‐kinase; SARS‐CoV‐2, severe acute respiratory syndrome coronavirus 2; STAT, signal transducer and activator of transcription.

Our therapeutic strategy differs fundamentally from that of the World Health Organization (WHO).^18^ We believe that the medicines recommended by the WHO, in addition to being very expensive, are also insufficiently effective for two reasons: (1) they inhibit viral replication, and this is not directly related to the hyperactivation of the NLRP3 inflammasome^18^, ^19^, ^20^, ^21^ and (2) it does not eliminate the cause of the CS, but trying to treat its effects (Figure 2). Some of them have a number of side effects, incl. potential mutagenicity for the host^22^ and death.^23^

WHO recommends medications that inhibit severe acute respiratory syndrome coronavirus 2 (SARS‐CoV‐2) replication, the interleukin‐6 receptor, and the tyrosine kinase Janus kinase.^18^ Our treatment strategy targets the inhibition of SARS‐CoV‐2 entry into the cell and the NLRP3 inflammasome, as the source of the CS. In several publications, we have shown the dramatic effect of higher doses of colchicine in saving patients’ lives and lowering in‐hospital mortality.^6^, ^16^, ^17^, ^18^, ^24^, ^25^, ^26^, ^27^

### 4.2 The COVID‐19 therapeutic effect of colchicine increases with increasing doses

In the already described 452^6^ and the present 333 inpatients, a clear trend in mortality benefit has emerged with increasing doses of colchicine.

In the large‐scale and heavily advertised streamlined, randomized, controlled, open‐label trial RECOVERY, the effect of colchicine with a loading dose of 1.5 mg and a maintenance dose of 1 mg daily on COVID‐19 inpatients was completely negated: no reductions in 28‐day mortality, duration of hospital stay, risk of progressing to invasive mechanical ventilation, or death. The study authors did not form a higher‐dose colchicine group similar to Terkeltaub et al.,^7^ although they surveyed more than 11,000 inpatients.^28^ And as for their conclusion, we completely agree; however, we maintain that their results are solely due to the low doses of colchicine they used.

Authors using slightly higher doses of colchicine found positive trends. For example, beginning with a loading dose of 2 mg and a maintenance dose of 1 mg per day has been reported to decrease mortality by Alsultan et al.^29^ as well as a significantly improved time to clinical deterioration.^30^

When doses are increased to 1.5 mg daily for 5 days, then 1 mg for a further 5 days, colchicine reduced the length of supplemental oxygen therapy and hospitalization and probably the mortality of COVID‐19.^31^

The use of colchicine is associated with a significant decrease in days of oxygen need, length of intensive care unit stays, less need for mechanical ventilation, accelerated recovery, and reduced mortality when the doses are 1.5 mg daily for 5 days, then 1 mg for 14 days.^32^

Karakaş et al. noted, even when comparing low doses of colchicine of 1 with 0.5 mg per day, beneficial effect of higher colchicine dosages.^33^

Increasing doses of colchicine positively enhances the treatment of COVID‐19, but the doses administered so far are far from effective.

In a randomized, open‐label trial, Perricone et al. applied the following regimen with colchicine—1.5 mg/day if weight was less than 100 kg or 2 mg/day if weight was more than 100 kg for a maximum of 30 days or until hospital discharge. They found no beneficial effect of colchicine on the rate and the time to the critical stage or 28‐day mortality.^5^ The authors explained the apparently conflicting results with other promising studies with that less severe^30^ or younger^31^ patients are included.

Perhaps the explanation for these differences is that colchicine per kg body weight is not taken into account. It is very likely that the younger Brazilian patients in the Lopes et al. study were also of lower weight, which automatically increases the dose of colchicine per kg.^31^ It should be expected that in developing countries^29^, ^31^, ^32^ patients have a lower body mass index index than those in Europe or the United States, Canada, and Australia, thus a better effect of colchicine should be expected.

We consider it a valid approach to try to differentiate patients by weight, but this difference so far is cosmetic.^5^ Should a patient over 100 kg be given a loading colchicine dose of 5 mg^17^ instead of 2 mg^5^ and then the effect would be dramatic.^6^, ^17^

Recently we described a unique effect of a wrongly taken overdose of colchicine (15 mg over 10 h) on a COVID‐19 inpatient with bilateral pneumonia and pericardial effusion. After the cessation of all other therapy, this single overdose proved sufficient to recover the patient, who was discharged on the ninth day. Two similar cases with 12 mg of colchicine were also presented and this overdose of colchicine was sufficient for the complete recovery of the two outpatients.^27^ In another case, a 120 kg inpatient with type 2 diabetes mellitus, hypertension, and gout despite the standard treatment, deteriorated sharply and this negative development of the disease was interrupted with a loading dose of 6 mg colchicine 26. These cases demonstrate the life‐saving effect of high but safe doses of colchicine in high‐risk COVID‐19 patients. In conclusion, high colchicine doses may play a major role and have a dramatic effect in the treatment of COVID‐19 patients.^17^, ^18^, ^24^, ^25^, ^26^

### 4.3 Mortality and bias discussion

The data we have used have been verified and corroborated by the hospital centers in question. When analyzing mortality by age, a potential source of bias was highlighted, namely that mortality was highest in the age group 70–74, which corresponded to the average life expectancy in the country (75.1 years for Bulgaria).^34^ We attempted to address this source of bias by separately calculating the average duration of hospital stay for both colchicine and control group, as well as analyzing the saturation levels of different groups. Mejia et al. showed that saturation levels at admission and during stay are good predictors of mortality.^35^ This correlation is highly accurate and has been used in models to predict mortality by Yadaw et al.,^36^ which is why we thought it prudent to include in the analysis.

The values obtained by us may be similar; however, the difference is statistically significant, indicating that colchicine may have contributed to a lower mortality in the active group. Deeper analysis of O_2_ levels at admission and lowest recorded levels during stay would definitively show a link between colchicine and lower mortality, which is lacking in our analysis and should be noted as a limitation of the study.

We posit that the addition of colchicine prolonged the hospital stay, especially for critical patients such as all which require hospitalization, thus helping control the symptoms and ultimately saving them. At the same time, this allowed for a controlled return to normal saturation levels helping patients survive the infection. It should be noted that this effect seems to be dose dependent, as shown in the 2 versus 4 mg comparison.

### 4.4 About the safety of the doses of colchicine we used

The higher doses of colchicine we used raises the question of the safety of our loading doses. We analyzed the deaths often cited in the literature with colchicine doses of 7^37^ and 7.5 mg^38^ and show that they are due to drug interactions.^7^ Doses of 15–18 mg of colchicine may cause serious side effects rather than death.^16^, ^39^, ^40^

Doses of colchicine below 0.1 mg/kg are completely safe,^41^ and those between 0.1 and 0.2 mg/kg may lead to intoxication in some cases, but not to death.^16^ In a retrospective study for a period of 10 years, the lowest lethal dose was 0.63 mg/kg.^42^ This is 14 times our maximum dose of colchicine (0.045 mg/kg).

Colchicine has repeatedly been administered in the so‐called loading “overdoses” from 3 to 6.7 mg without any life‐threatening effect.^7^, ^16^, ^43^, ^44^, ^45^, ^46^, ^47^ Moreover, in cases of familial Mediterranean fever (FMF) resistance, a colchicine “maximum tolerated dose” is recommended.^43^ The effect of high‐dose colchicine in COVID‐19 obese patients^26^, ^48^ and the effect of accidental colchicine overdoses in a COVID‐19 patients^27^ are in line with this opinion.

The most common adverse drug reactions, related to colchicine, are gastrointestinal, in particular diarrhea. In the study, approximately 48% of patients developed diarrhea, which is similar to the adverse drug reactions reported in the literature.^7^

BHH earliest possible application is crucial for its effect. BHH is most effective when given prophylactically or started by inhalation after contact with COVID‐19 carrier. When the first symptoms of COVID‐19 appear, the viral load is already at its maximum, which limits the effect of bromhexine. In hospital conditions, inhalations with BHH will be useful, although there, in the first place, the question of inhibition of NLRP3 inflammasome is leading with Figure 2 showing the proposed mechanism of inhibition.^18^, ^48^

### 4.5 Different doses of colchicine for different pathological conditions

One of the variants of Paracelsus' golden rule is “All drugs are poisons; the benefit depends on the dosage.”^49^

Colchicine is a typical example of dose manipulation. Optimal doses for different pathological conditions are different. The cardioprotective effects of colchicine are well known and might be due at least to its ability to inhibit the pro‐thrombotic activity of oxLDL^50^ and leukocyte‐platelet aggregation.^51^, ^52^ The doses recommended for pericarditis, atrial fibrillation, stroke prevention, vascular inflammation prevention, myocardial infarction, acute coronary syndrome coronary artery disease, cardioprotection are within the limits of 0.5–1 mg daily. Rarely the loading doses reach 2 mg.^53^ Early treatment with low colchicine dose reduced high‐sensitivity C‐reactive protein levels in the patients with acute minor ischemic stroke and transient ischemic attack.^54^ In cases of acute gout, the loading dose is 1.5–1.8 mg and those of FMF reaches to 2.4 mg.^7^, ^43^, ^53^ However, as we demonstrated higher doses of colchicine (up to 5 mg loading dose) are required to inhibit the COVID‐19 CS.^6^, ^18^, ^24^, ^25^, ^26^, ^27^, ^48^

### 4.6 Perspectives

A large number of viruses can overactivate the NLRP3 inflammasome and we are convinced that higher colchicine doses would be useful in these cases, as well.^48^ We set out to test this hypothesis with success.

## 5 CONCLUSION

Colchicine can safely be used to treat COVID‐19 outpatients/inpatients in the mentioned doses, provided that liver and kidney damage are not present and adverse drug interactions are avoided.

## AUTHOR CONTRIBUTIONS

**Rumen Tiholov**: Data curation; formal analysis. **Aleksander I. Lilov**: Data curation; investigation. **Gergana Georgieva**: Data curation; investigation. **Kiril R. Palaveev**: Data curation; formal analysis. **Konstantin Tashkov**: Software. **Vanyo Mitev**: Methodology; project administration; resources; supervision; visualization; writing—original draft; writing—review and editing.
